# Supplementary material for: Cross-border climate vulnerabilities of the European Union to drought
Source: Nat Commun. 2021 Jun 15;12:3322. doi: 10.1038/s41467-021-23584-0 (PMC8206334; doi:10.1038/s41467-021-23584-0)
Supplement: Supplementary file 1 — Supplementary Information [file 41467_2021_23584_MOESM1_ESM.pdf]

Supplementary Information
Cross-border climate vulnerabilities of the European Union to drought

Ertug Ercin [a,b], Ted I. E. Veldkamp[c], Johannes Hunink[d]

a R2Water Research and Consultancy, Amsterdam, the Netherlands
b Institute for Environmental Studies, Vrije Universiteit Amsterdam, the Netherlands
c Amsterdam University of Applied Science, Amsterdam, the Netherlands
d FutureWater, Cartagena, Spain

*Correspondence: ercin@r2water.nl; Tel.: +31617366 472

1.1 Supplementary Tables

Table S.1: Population forecasts used in the analysis, SSP2, in millions

Country	Unit	2030	2050	2085
Austria	million	8.946	9.214	8.717
Belgium	million	11.775	12.596	13.156
Bulgaria	million	6.754	6.308	5.485
Croatia	million	4.259	4.063	3.523
Cyprus	million	1.377	1.579	1.630
Czech Republic	million	11.292	11.704	11.325
Denmark	million	6.087	6.574	7.296
Estonia	million	1.297	1.259	1.176
Finland	million	5.845	6.176	6.740
France	million	70.324	76.504	82.991
Germany	million	81.357	78.932	71.267
Greece	million	11.379	11.248	10.093
Hungary	million	9.488	8.899	7.512
Ireland	million	5.513	6.358	7.047
Italy	million	61.954	61.476	55.413
Latvia	million	2.020	1.835	1.488
Lithuania	million	3.051	2.713	2.145
Luxembourg	million	0.676	0.831	0.968
Malta	million	0.444	0.443	0.402
Netherlands	million	17.884	18.575	19.223
Poland	million	37.889	35.163	28.619
Portugal	million	11.082	11.310	10.722
Romania	million	19.915	17.757	12.800
Slovakia	million	5.669	5.530	4.782
Slovenia	million	2.135	2.199	2.196
Spain	million	50.208	52.760	50.331
Sweden	million	10.986	12.512	14.784
United Kingdom	million	70.037	76.592	83.856
Total		529.6442	541.1092	525.6878

Table S.2: Calculated annual growth rate of imports by the EU per crop group for 2030, 2050 and 2085 under SSP2 characteristics.

Annual grow rate of imports, in %	2010-2030	2030 - 2050	2050-2085
Cereals	0.90	-1.74	-0.56
Fibre crops	0.89	-0.13	-0.14
Oil crops	0.70	0.02	-0.13
Pulses	1.40	-0.16	-0.16
Roots	-0.74	-7.57	-9.38
Sugar crops	3.22	-0.89	-0.15
Vegetables and fruit	-0.57	-0.50	0.03
Other crops	-1.30	0.09	0.04

1.2 Supplementary Figures

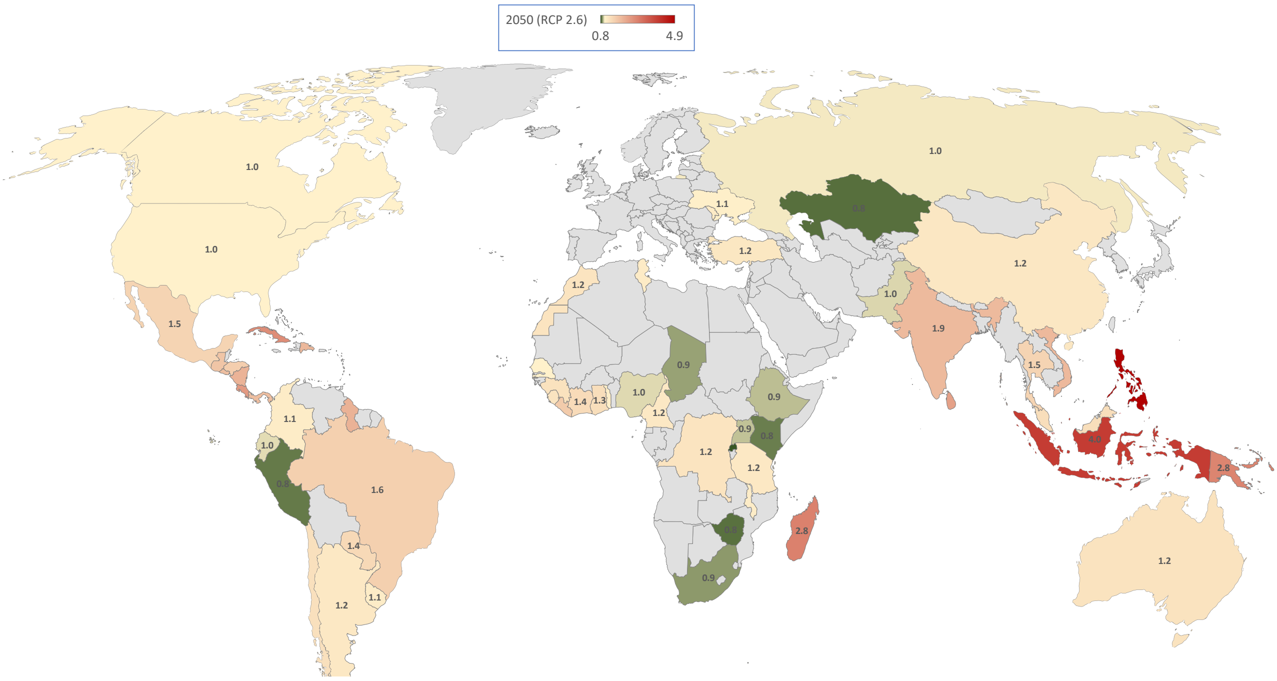

Figure S.1: Cross-border climate vulnerability score (CCVS) of the EU's agri-food economy to drought per exporting country in 2050 under the RCP 2.6 concentration pathway. Green to red colours indicating the CCVS in ascending order.
