# Supplementary material for: Cross-border climate vulnerabilities of the European Union to drought
Source: Nat Commun. 2021 Jun 15;12:3322. doi: 10.1038/s41467-021-23584-0 (PMC8206334; doi:10.1038/s41467-021-23584-0)
Supplement: Supplementary file 3 — Reporting Summary [file 41467_2021_23584_MOESM3_ESM.pdf]

nature research

Corresponding author(s): NCOMMS-20-12637A

Last updated by author(s): Mar 22, 2021

Reporting Summary

Nature Research wishes to improve the reproducibility of the work that we publish. This form provides structure for consistency and transparency in reporting. For further information on Nature Research policies, see Authors & Referees and the Editorial Policy Checklist .

Statistics

For all statistical analyses, confirm that the following items are present in the figure legend, table legend, main text, or Methods section.

n/a	Confirmed	
☒	☐	The exact sample size (*n*) for each experimental group/condition, given as a discrete number and unit of measurement
☒	☐	A statement on whether measurements were taken from distinct samples or whether the same sample was measured repeatedly
☒	☐	The statistical test(s) used AND whether they are one- or two-sided<br>*Only common tests should be described solely by name; describe more complex techniques in the Methods section.*
☒	☐	A description of all covariates tested
☒	☐	A description of any assumptions or corrections, such as tests of normality and adjustment for multiple comparisons
☐	☒	A full description of the statistical parameters including central tendency (e.g. means) or other basic estimates (e.g. regression coefficient) AND variation (e.g. standard deviation) or associated estimates of uncertainty (e.g. confidence intervals)
☒	☐	For null hypothesis testing, the test statistic (e.g. *F*, *t*, *r*) with confidence intervals, effect sizes, degrees of freedom and *P* value noted<br>*Give P values as exact values whenever suitable.*
☒	☐	For Bayesian analysis, information on the choice of priors and Markov chain Monte Carlo settings
☒	☐	For hierarchical and complex designs, identification of the appropriate level for tests and full reporting of outcomes
☒	☐	Estimates of effect sizes (e.g. Cohen's *d*, Pearson's *r*), indicating how they were calculated

Our web collection on statistics for biologists contains articles on many of the points above.

Software and code

Policy information about availability of computer code

Data collection	R-3.6.3 software , QGIS 3.10.3, Matlab R2019a
Data analysis	R-3.6.3 software , QGIS 3.10.3, Excel for Mac, Matlab R2019a

For manuscripts utilizing custom algorithms or software that are central to the research but not yet described in published literature, software must be made available to editors/reviewers. We strongly encourage code deposition in a community repository (e.g. GitHub). See the Nature Research guidelines for submitting code & software for further information.

Data

Policy information about availability of data

All manuscripts must include a data availability statement. This statement should provide the following information, where applicable:

- Accession codes, unique identifiers, or web links for publicly available datasets
- A list of figures that have associated raw data
- A description of any restrictions on data availability

The datasets generated during and/or analysed during the current study are available from the corresponding author on reasonable request.

Field-specific reporting

Please select the one below that is the best fit for your research. If you are not sure, read the appropriate sections before making your selection.

☐ Life sciences ☐ Behavioural & social sciences ☒ Ecological, evolutionary & environmental sciences

For a reference copy of the document with all sections, see nature.com/documents/nr-reporting-summary-flat.pdf

Ecological, evolutionary & environmental sciences study design

All studies must disclose on these points even when the disclosure is negative.

Study description	We quantified and mapped cross-border climate vulnerabilities of the EU's agri-food economy in relation to drought severity in third countries . To quantify and map them, we first calculated dependency of the EU on the external rainfall, green virtual water imports, by the target years 2030, 2050 and 2085 using Shared Socio-economic Pathways 2 (SSP2) characteristics. In the next step, we calculated how drought severity changes in exporting locations under different climate change scenarios (under Representative Concentration Pathway 2.6 and 6.0). Combining green virtual water import volumes with projected drought severity provides cross-border climate vulnerabilities of the EU's agri-food economy to drought, expressed in terms of a score calculated as a change in vulnerabilities under different climatic conditions. Data resources, analysis and description of the method is given in Method and Data section.
Research sample	Data used for the drought analysis is hydrological data generated as part of the ISIMIP 2b simulation. To perform the drougth analysis we used an ensemble of 4 general circulation models in combination with 4 global hydrological models. 2 climate scenario's were studied in the manuscript by using the Representative Concentration Pathways RCP2.6 and RCP6.0. Model outputs were converted into groups of statistics and we used the median values to present our results.
Sampling strategy	The ensemble of 4 general circulation models in combination with 4 global hydrological models have a proven scientific track-record to be a representative sample to be used in climate impacts studies focusing on consequences for water availability, and drought.
Data collection	Hydrological data for the full ensemble was obtained from ISIMIP (www.isimip.org) and processed and analysed by the author and co-author.
Timing and spatial scale	Hydrological data used within this manuscript is produced on a 0.5x0.5 degree grid scale and with a monthly temporal resolution. As part of the analysis data has been resampled to yearly statistics.
Data exclusions	No data was excluded from analysis
Reproducibility	Methodology of production of hydrological data was described in previous scientific literature. An overview of the modelling set-up is presented on: https://www.isimip.org/protocol/#isimip2b. Methodology applied to process and analyse the data was presented in the methods section of the manuscript.
Randomization	n.a.
Blinding	n.a.

Did the study involve field work? ☐ Yes ☒ No

Reporting for specific materials, systems and methods

We require information from authors about some types of materials, experimental systems and methods used in many studies. Here, indicate whether each material, system or method listed is relevant to your study. If you are not sure if a list item applies to your research, read the appropriate section before selecting a response.

Materials & experimental systems

n/a	Involved in the study	
☒	☐	Antibodies
☒	☐	Eukaryotic cell lines
☒	☐	Palaeontology
☒	☐	Animals and other organisms
☒	☐	Human research participants
☒	☐	Clinical data

Methods

n/a	Involved in the study	
☒	☐	ChIP-seq
☒	☐	Flow cytometry
☒	☐	MRI-based neuroimaging
